# Automatic segmentation of stereoelectroencephalography (SEEG) electrodes post-implantation considering bending

**DOI:** 10.1007/s11548-018-1740-8

**Published:** 2018-05-07

**Authors:** Alejandro Granados, Vejay Vakharia, Roman Rodionov, Martin Schweiger, Sjoerd B. Vos, Aidan G. O’Keeffe, Kuo Li, Chengyuan Wu, Anna Miserocchi, Andrew W. McEvoy, Matthew J. Clarkson, John S. Duncan, Rachel Sparks, Sébastien Ourselin

**Affiliations:** 1 Wellcome/EPSRC Centre for Interventional and Surgical Sciences, UCL, London, UK; 2 National Hospital for Neurology and Neurosurgery, London, UK; 3 Department of Clinical and Experimental Epilepsy, Institute of Neurology, National Hospital for Neurology and Neurosurgery, London, UK; 4 Department of Statistical Science, University College London, London, UK; 5 The First Affiliated Hospital of Xian Jiaotong University, Xian, People’s Republic of China; 6 Vickie and Jack Farber Inst for Neuroscience, Thomas Jefferson University, Philadelphia, USA; 7 Dementia Research Centre, Department of Neurodegenerative Disease, UCL Institute of Neurology, London, UK

**Keywords:** Epilepsy, SEEG, Automatic segmentation, Bending

## Abstract

**Purpose:**

The accurate and automatic localisation of SEEG electrodes is crucial for determining the location of epileptic seizure onset. We propose an algorithm for the automatic segmentation of electrode bolts and contacts that accounts for electrode bending in relation to regional brain anatomy.

**Methods:**

Co-registered post-implantation CT, pre-implantation MRI, and brain parcellation images are used to create regions of interest to automatically segment bolts and contacts. Contact search strategy is based on the direction of the bolt with distance and angle constraints, in addition to post-processing steps that assign remaining contacts and predict contact position. We measured the accuracy of contact position, bolt angle, and anatomical region at the tip of the electrode in 23 post-SEEG cases comprising two different surgical approaches when placing a guiding stylet close to and far from target point. Local and global bending are computed when modelling electrodes as elastic rods.

**Results:**

Our approach executed on average in 36.17 s with a sensitivity of 98.81% and a positive predictive value (PPV) of 95.01%. Compared to manual segmentation, the position of contacts had a mean absolute error of 0.38 mm and the mean bolt angle difference of $0.59^{\circ }$ resulted in a mean displacement error of 0.68 mm at the tip of the electrode. Anatomical regions at the tip of the electrode were in strong concordance with those selected manually by neurosurgeons, $ICC(3,k)=0.76$, with average distance between regions of 0.82 mm when in disagreement. Our approach performed equally in two surgical approaches regardless of the amount of electrode bending.

**Conclusion:**

We present a method robust to electrode bending that can accurately segment contact positions and bolt orientation. The techniques presented in this paper will allow further characterisation of bending within different brain regions.

**Electronic supplementary material:**

The online version of this article (10.1007/s11548-018-1740-8) contains supplementary material, which is available to authorized users.

## Introduction

Epilepsy is a disease characterised by an enduring predisposition to generate epileptic seizures and affects 1% of the population [8]. A third of patients develop chronic refractory focal epilepsy and neurosurgery may provide a cure [9].

Brain imaging is fundamental in a typical neurosurgical evaluation for determining the epileptogenic zone (EZ) with modalities including structural and functional MRI (e.g. T1/T2-w, FLAIR) and PET [9]. If the EZ is not identifiable, invasive electroencephalography (EEG) recordings are performed in the form of stereo-EEG (SEEG) or subdural grid insertion. SEEG is a procedure in which multiple electrodes are stereotactically inserted to identify the seizure onset zone [21]. Accurate placement of electrode contacts is important for safety, interpretation of the recorded electrical signals, and subsequent resection planning [21]. Planning of electrode implantation is crucial for avoiding blood vessel damage and subsequent intracranial haemorrhage (which occurs in 1–2% of patients), and automatic computer-assisted multiple trajectory planning tools have been proposed [17, 18]. However, intraoperatively, entry point (EP) accuracy can be affected by misregistration of the neuronavigation system, inaccurate alignment, and deflection during drilling, whereas target point (TP) errors may be caused by the angle at which the electrode passes through skull, deflection of the electrode at the dura or within the brain, the rigidity of the electrode, and the depth to which a guiding stylet is inserted [3, 21]. Robotic systems have been introduced to improve EP implantation accuracy [3, 7]. However, TP displacement is the main source of error and understanding why and how electrodes bend may help predict final TP positions during surgical planning and improve EZ localisation [22].

Furthermore, it is convenient to have a rapid and reliable scheme for segmenting contacts, assigning their anatomical location when interpreting SEEG studies and for guiding definitive surgical resections. Automatic segmentation approaches have been proposed for SEEG [2, 14, 16] and deep brain stimulation (DBS) [5, 10, 11] implantation. Arnulfo et al. [2] used post- implantation CT (threshold = 1600) co-registered with MRI to segment electrodes based on a geometrical-constrained search. They randomly generated different scenarios for 1–15-mm displaced TP in an experimental study and reported accuracy of 10% of false negatives (FN) and 7% of false positives (FP) for a maximum displacement of 15 mm. However, bending may occur at any point along the electrode’s trajectory. Meesters et al. [14] co-registered the CT (threshold = 500 HU) with MRI and extracted guiding screws with a multi-scale filter whilst determining likely tip locations within a wedge-shape region. However, manual adjustments took between 10 s and several minutes, and reported deviations of the tip and their method did not account for electrodes bending. Additionally, these methods relied on pre-operative plans and were tested only on one electrode type.

Hubsch et al. [10, 11] proposed an automated algorithm reconstructing full electrode trajectory whilst accounting for DBS electrode bending from CT scans. A convex hull brain mask is extracted using thresholds, and the largest connected components are skeletonised [10]. Trajectories of 11 electrodes are modelled fitting a polynomial function and then aligned to a common coordinate system reporting mean deviation that varies from 0.92 to 2.0 mm. However, they have mostly focused on fitting trajectories using polynomials rather than computing the amount of electrode bending and have not considered the reasons of bending within the brain anatomy. Although Lalys et al. [13] looked at the reasons of bending (mainly due to brain shift) by computing a local and mean curvature index over the entire length of DBS electrodes, the index provides no information about the direction of bending. Unlike SEEG procedures, where 8–14 electrodes are inserted, DBS electrodes are typically inserted bilaterally and the contacts are very close to the tip. To discriminate between contacts located in white or grey matter, Arnulfo et al. [1, 16] compute the distance from each contact to grey–white matter interface.

## Contribution of this paper

**Fig. 1:**
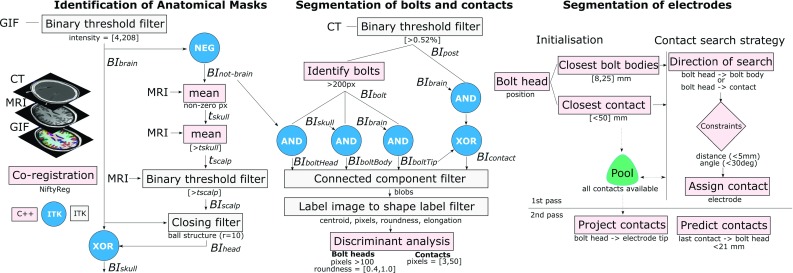
Flow chart of algorithm pipeline

Our main motivation is to automatically segment SEEG contacts and bolts (Ad-Tech Med Instr Corp, USA) relative to the anatomy whilst accounting for electrode bending along its trajectory at contact positions rather than as a result of TP displacement. Our algorithm (Fig. 1) allows estimating not only the position of contacts but also the direction of the bolts inserted into the skull since the angle of the bolt with respect to the scalp surface normal is a measure of post-implantation accuracy. We quantify local and global bending by means of electrodes modelled as elastic rods in position-based dynamics^1^ and validate our methods in 23 post-SEEG cases (224 electrodes, 1843 contacts) comprising two different surgical approaches (placing a guiding stylet close to or far from the TP).

## Methods

### Input images

**Fig. 2:**
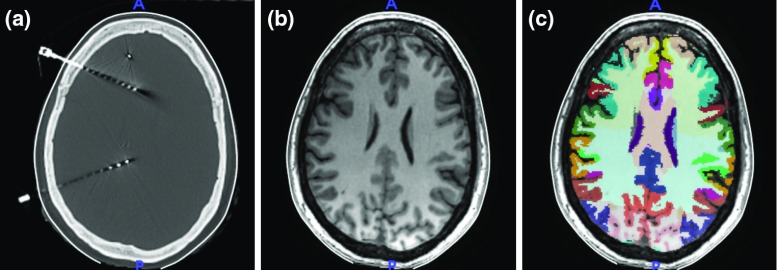
Input images: **a** post-SEEG implantation CT, **b** MRI T1, and **c** parcellation

A post-SEEG implantation resampled CT and an MRI T1 images are rigidly co-registered using NiftyReg (v1.5.43) [15]. From the MRI image, we obtain the parcellation of brain anatomy via NiftyWeb (GIF v3.0) (Fig. 2) [4].

**Fig. 3:**
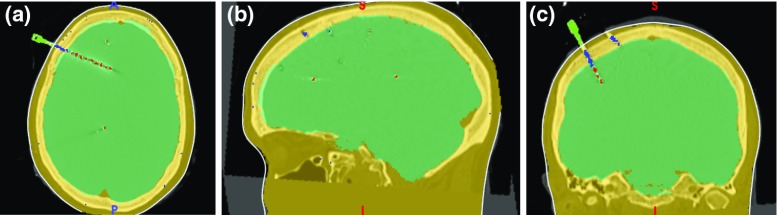
**a** Axial, **b** sagittal and **c** coronal planes showing computed masks of the brain (cyan), skull and scalp (yellow) together with the result of connected components filters of contacts (red), bolt head (green), and the section of the bolt crossing the skull (blue)

### Identification of anatomical masks

We use the MRI and the parcellation to create regions of interest that are used to identify contacts, bolt heads, and the section of the bolt crossing the scalp/skull, which we refer as *bolt body*. First, a BinaryThresholdImageFilter is applied to the parcellation to create a mask of intracranial space $BI_{\mathrm{brain}}$, i.e. with a threshold $t_{\mathrm{brain}}$ in the range of $4 \le t_{\mathrm{brain}} \le 208$. We apply a method similar to Dogdas et al. [6], which we describe herein for completeness. We compute a skull threshold $t_{\mathrm{skull}}$ from the MRI as the mean of the intensities of the nonzero voxels that are not brain as an empirical measure to split the low- and high-intensity regions, followed by a scalp threshold $t_{\mathrm{scalp}}$ as the mean of the non-brain voxels above the skull threshold ($\forall I_{MRI}(x,y,z)\ge {t_{MRI_{\mathrm{skull}}}}$) to identify the transition between the head and the background.

**Table 1:** Geometrical analysis, $\mu (\sigma )$, and discriminant analysis of bolt heads and contacts

	Geometrical analysis			Discriminant analysis	
	Number of Pixels	Elongation	Roundness	Number of pixels	Roundness
Bolt head	329.4 (183.5)	2.51 (0.59)	0.63 (0.06)	> 100	[0.4, 1.0]
Contact	9.7 (6.6)	2.52 (1.27)	1.10 (0.06)	[3, 50]	

**Fig. 4:**
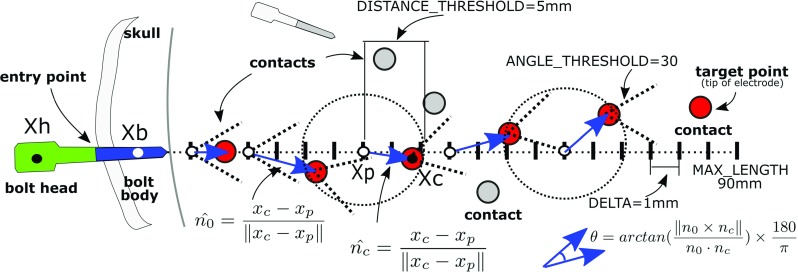
Search strategy given the direction of the bolt and constraints (distance and angle)

A BinaryThresholdImageFilter is applied to the MRI to create a mask of the scalp $BI_{\mathrm{scalp}}$ with a lower threshold equal to $t_{\mathrm{scalp}}$. We use morphological operators to combine $BI_{\mathrm{brain}}$ and $BI_{\mathrm{scalp}}$ and apply a closing filter with a ball structuring element (radius = 10) to obtain a mask of the head, i.e. $BI_{\mathrm{head}}=(BI_{\mathrm{scalp}} \cup BI_{\mathrm{brain}}) \odot B_{10}$, and a mask of the skull, i.e. $BI_{\mathrm{skull}}=BI_{\mathrm{head}} \oplus BI_{\mathrm{brain}}$, after applying an XOR morphological operator on the result (Fig. 3).

### Segmentation of electrode bolts and contacts

A mask $BI_{\mathrm{post}}$ is created from a BinaryThresholdImageFilter applied to the post-op CT with lower threshold $t_{\mathrm{CT}}=(0.52)*max(I_{\mathrm{CT}}(x,y,z))$. $BI_{\mathrm{post}}$ is used to identify full bolts ($BI_{\mathrm{bolt}}$) with at least a minimum of 200 pixels. Three subsections are identified: the head of the bolt which is outside the patient’s head ($BI_{\mathrm{bolt}} \cap \lnot {BI_{\mathrm{head}}}$), the body ($BI_{\mathrm{bolt}} \cap BI_{\mathrm{skull}}$), i.e. section crossing the skull, and the tip ($BI_{\mathrm{bolt}} \cap BI_{\mathrm{brain}}$). Lastly, contacts are identified within the brain whilst excluding bolt tips ($(BI_{\mathrm{post}} \cap BI_{\mathrm{brain}}) \oplus BI_{\mathrm{boltTip}}$). We applied a ConnectedComponentImageFilter to the masks and a LabelImageToShapeLabelMapFilter to the blobs to get their centroids and geometrical properties before conducting geometrical analysis to identify discriminants of segmentation (Table 1). We detected contacts with blobs that were within a range of number of pixels ([3, 50]) and bolt heads with blobs that had a minimum number of pixels ($\ge 100$) and were within a range of roundness values ([0.4, 1.0]).

### Contact search strategy

Given a bolt head ($x_\mathrm{h}$) and its closest bolt body ($x_\mathrm{b}$) positions, we compute the direction of search ($\hat{n_0}=\frac{x_\mathrm{b}-x_\mathrm{h}}{\left||x_\mathrm{b}-x_\mathrm{h}\right||}$) and iteratively compute a number of points $x_p$ given a maximum electrode length (90 mm) and a step size (1 mm) in the direction $\hat{n_0}$. An available contact $x_\mathrm{c}$ is assigned to the electrode if and only if it is located below a distance constraint from $x_p$ (5 mm) and the angle between the previous direction $\hat{n_0}$ and the current direction $\hat{n_\mathrm{c}}$ is below an angle constraint ($30^{\circ }$) (Fig. 4), constraints which favour assigning contacts in the direction of the bolt during a first pass.

**Fig. 5:**
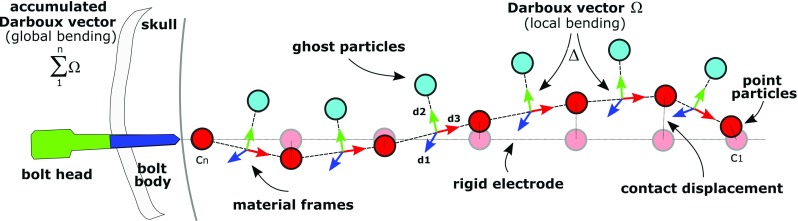
Modelling of electrodes as elastic rods. Bolt head (green) and body (blue) with contacts (red) modelled as point particles and ghost particles (cyan) created orthogonally along the electrode with material frames located between contacts

### Automatic segmentation of electrodes

The main steps of our algorithm include:

1. *Initialisation* All segmented contacts are initially labelled as ’available’ and stored in a pool. Given a bolt head position ($x_\mathrm{h}$), the closest bolt bodies ($8 \le \left||x_\mathrm{h}-x_\mathrm{b}\right|| \le 25$ mm) and the closest contact ($\left||x_\mathrm{h}-x_\mathrm{c}\right|| \le 50$ mm) are identified in order to narrow the search down to only those relevant.
2. *Contact search strategy* For each bolt head, the contact search strategy is executed initially with the closest bolt body (1st pass search) and subsequently with alternative bolt bodies if no contacts have been assigned. Although rare, bolt bodies may not be segmented and a direction of search cannot be computed. Therefore, the contact search strategy is called again with the closest contact position rather than a bolt body position.
3. *Project remaining contacts in pool* For electrodes containing at least one contact, we compute the minimum distance between an available contact in the pool and a line formed by the positions of the bolt head and the electrode tip. The contact is assigned to the electrode if and only if its distance to the closest point $x_\mathrm{p}$ to the line (tangent to the line) is below a constraint (5 mm) and $x_\mathrm{p}$ remains along the line or in a position of the line 20% extended from the tip, i.e. within an interpolation range of [0.0, 1.2] to project contacts that are further from the currently identified tip of the electrode.
4. *Predict contacts in the bolt region* For a given electrode, we compute the most common segment along the electrode based on the distances between subsequent contacts rounded to the closest integer. Based on electrode specification, we infer the type of electrode depending on the order of the segments and specify contact spacing. We then compute the direction from the last contact $x_{c_n}$ towards the bolt head $x_\mathrm{h}$ and create new contacts up to 21 mm before the bolt head position to segment only those contacts closer to the skull.

### Bending estimation

To quantify electrode bending, electrodes are modelled as elastic rods using the Cosserat model proposed by [19] and then implemented by [12] in position-based dynamics. Electrode contact positions are represented as linked particles with ghost particles located orthogonally half-way between contact pairs (Fig. 5). A material frame is created between contacts with a unit vector ($d_3=X_{c_{n-1}}-X_{c_n}$) aligned tangentially to its centreline followed by two additional orthonormal vectors, ($d_2=\hat{d_3} \times (X_{c_{n-1}}-X_{c_n})$) and ($d_1=\hat{d_2} \times \hat{d_3}$) chosen to lie in the principal direction of the cross section. We compute the rate of change of two consecutive frames, namely a Darboux vector $\Omega$, to describe local bending at the contact points [12, 20]. Along the electrode, $\Omega$ values are then accumulated to quantify global bending. We then use the parcellation to report the region at which each contact is located and report all those regions that the electrode passes through. Lastly, contact displacement and depth are estimated with respect to a rigid electrode with position of contacts projected along the direction from the bolt head to the last contact ($X_{c_n}$) at distances subject to electrode specification.

**Fig. 6:**
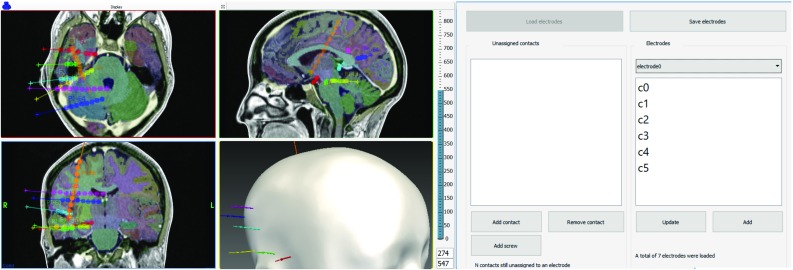
Automatic segmentation of electrodes interface and GUI for manual adjustment

### Validation

We asked two neurosurgeons and one clinical scientist to (a) manually segment the contacts of a random subset of electrodes ($N=109$ contacts), (b) manually identify the tip and head of the bolt of a random subset of electrodes ($N=95$ bolts), (c) confirm the correct number and location of contacts and electrodes ($N=23$ cases), and (d) identify the TP anatomical region ($N=222$ electrodes).

**Fig. 7:**
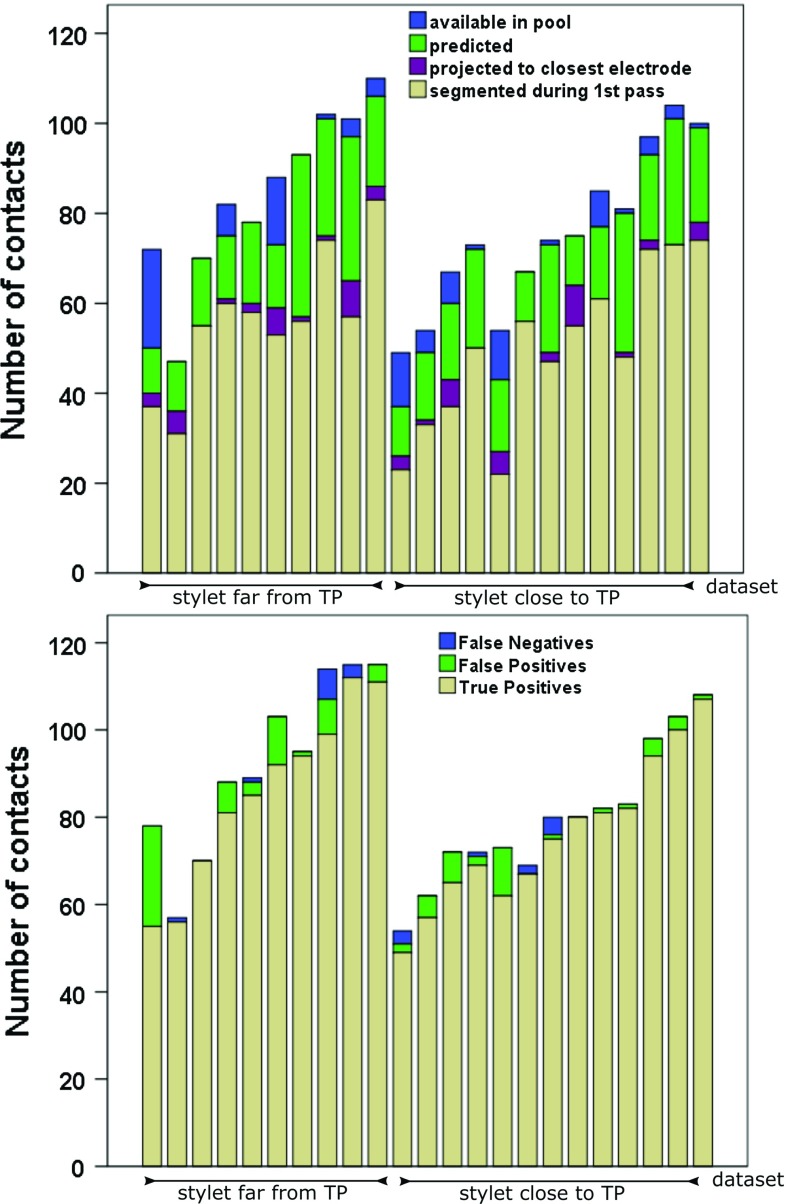
**Top:** Number of contacts initially segmented and assigned to electrodes via a bolt head and bolt bodies association (step 2), projected (step 3), predicted (step 4), and left unassigned and available in pool. **Bottom:** Number of contacts correctly segmented (TrP—true positives), wrongly segmented (FP) and missed (FN) in 23 data sets (order by number of electrodes)

## Results

### Interface

We implemented our algorithms in C++ using MITK^2^ and ITK^3^ as well as a GUI in Qt to allow clinicians to adjust the automatic segmentation if needed (Fig. 6). On average, our method executed in 36.17 s ($N=23$, $\sigma =15.7$), faster than manual segmentation.

### Performance

**Fig. 8:**
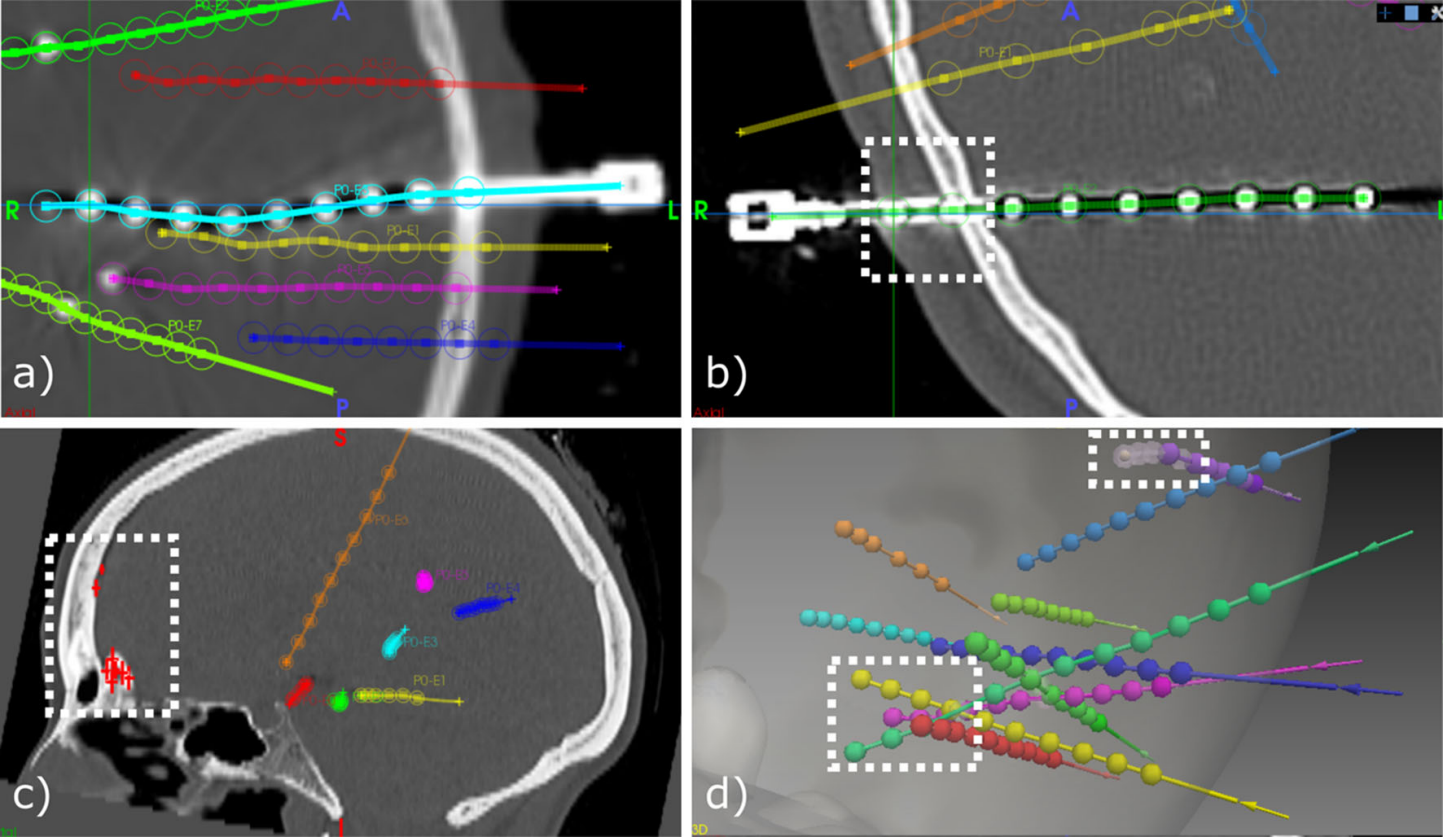
Examples: **a** segmented bolt head and contacts of electrodes overlaying CT; **b** contacts predicted at the skull and scalp level; **c** 22 FP (red marks along the skull); and **d** our worst case with 3 contacts not segmented due to crossings and 4 FNs

Of a total of 224 electrodes (1843 contacts), 29 contacts were segmented but not assigned to any electrode due to: (a) three bolt heads that were not automatically segmented (17), (b) no segmented contacts close to them (5), and (c) due to one incorrectly assigned contact to a bolt head (7). On average, the sensitivity ($\frac{TrP}{TrP+FN}*100$) and PPV ($\frac{TrP}{TrP+FP}*100$) of our approach was $\mu =98.81\%$; $\sigma =2.04$ (false-negative rate of $\mu =0.124$; $\sigma =0.02$) and $\mu =95.01\%$; $\sigma =6.73$ (false- positive rate of $\mu =0.059$; $\sigma =0.09$), respectively (Fig. 7 bottom), finding no statistical significant difference between data sets of the two surgical approaches, i.e. placing a stylet far from or close to target point. To illustrate our results, Fig. 8 shows two cases correctly identified (a, b) along two worst cases (c, d).

### Validation

Computed contact positions, bolt angles, and regions of anatomy are compared with those manually segmented in a subset of cases (Table 2).

**Fig. 9:**
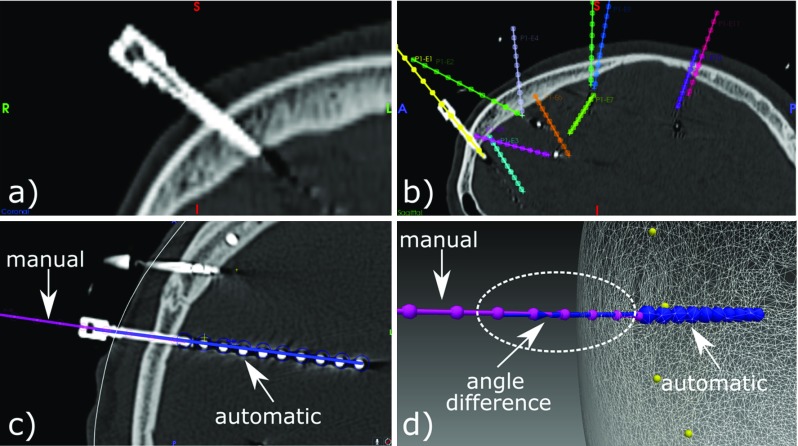
Bolt angles. **a** Bolt from post-CT image and **b** manual identification of the direction along bolts by a clinical scientist. **c**, **d** (Inconspicuous) comparison of manual (pink) and automatic identification (rigid electrode shown in blue) of bolt direction of an outlier case with angle difference of $\theta _{\mathrm{error}}=5.73^{\circ }$ and displacement error at the tip of $d_{\mathrm{error}}=4.79$ mm

**Fig. 10:**
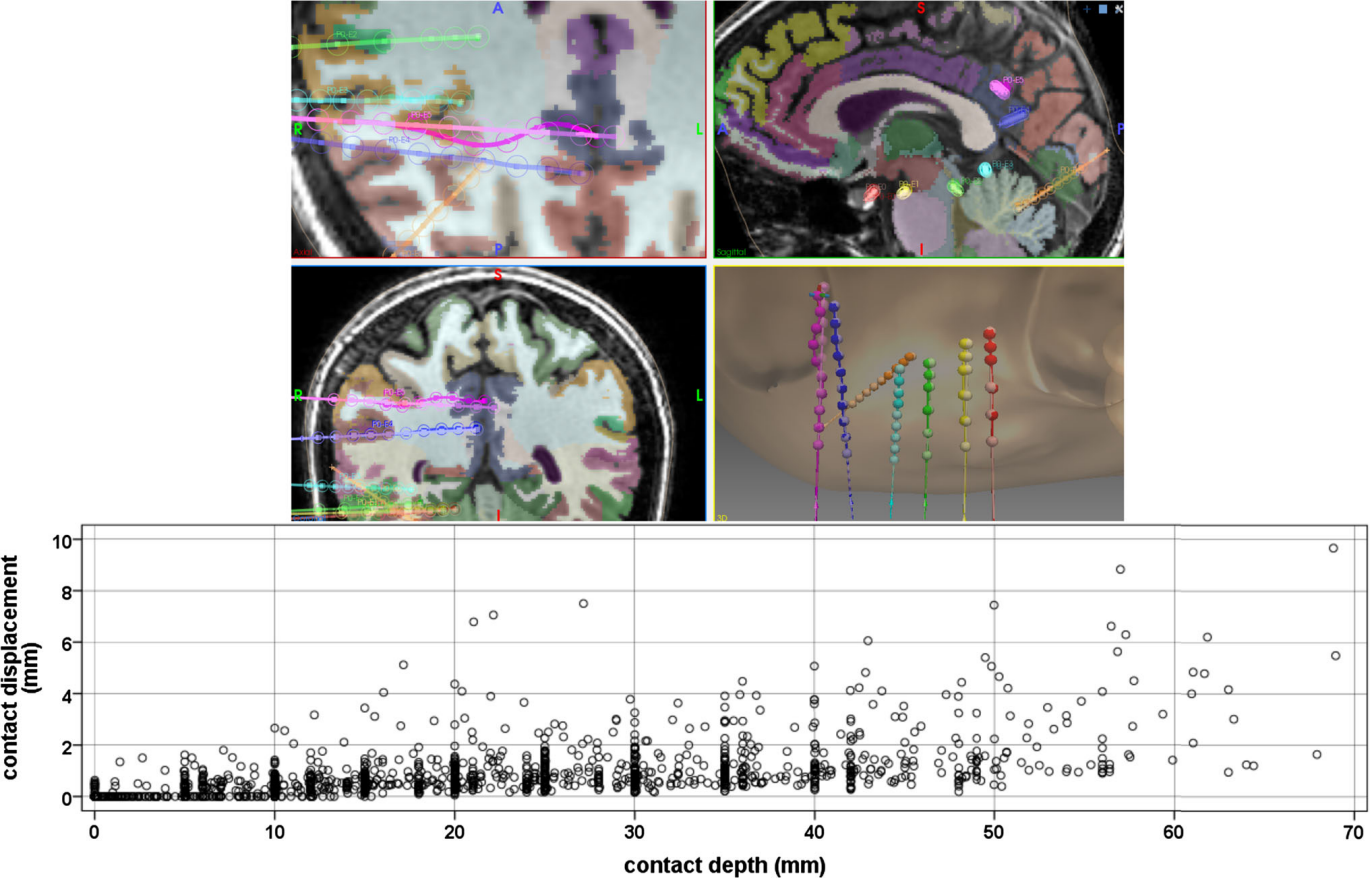
Anatomical regions traversal (top) and contact displacement (bottom) of automatically segmented electrodes with respect to a rigid electrode computed based on bolt direction

- *Contact position* Compared to the manual segmentation done by a clinical scientist (M1) and a neurosurgeon (M2), we found that the contact location of our automatic segmentation approach had a mean absolute error (MAE) of 0.38 and 0.40 mm, respectively, and a root-mean-square deviation (RMSD) of 0.45. The distance of contact positions between both manual segmentations was on average $\mu =0.37$ mm ($\sigma =0.22$). We found no statistical difference when comparing the distances from automatically computed contact position to those positions obtained via manual segmentation (paired differences: $\mu =0.036$, $\sigma =0.21$).
- *Bolt angle* We found that the angle of bolts between automatic and manual segmentation (Fig. 9) by M1 and M2 differed on average by $0.59^{\circ }$ and $0.22^{\circ }$, respectively, with pair samples strongly and positively correlated (Pearson correlation) and with strong reliability (Cronbach’s alpha). We study the displacement error $d_{\mathrm{error}}=\mathrm{sin}(\theta _{\mathrm{error}})*l_\mathrm{e}$ at the tip of the electrode caused by this angle difference $\theta _{\mathrm{error}}$ and the length of the electrode $l_\mathrm{e}$ within the brain and define a maximum tolerance value $T_e=2.29$ mm related to contact length. On average, $d_{\mathrm{error}}$ at the tip of a rigid electrode caused by the angle difference is $\mu =0.68$ mm for M1 and $\mu =0.72$ mm for M2. We found 3 outliers above $T_e$ for M1 ([2.46, 4.79] mm) and 8 outliers for M2 ([2.37, 5.48] mm). Given $T_e$, a non-inferiority test indicates that 0.68 mm is an estimate of $d_{\mathrm{error}}$ with 95% of CI (0.431–0.926) after accounting for clustering using a patient-level random effect. Figure 10 shows an example with electrodes automatically segmented and their corresponding rigid electrodes (lighter colours) computed using the direction of bolts automatically segmented.
- *Regions of anatomy* We also ran a intra-class correlation two-way mixed effects model with average measures and found a strong agreement when identifying the anatomical region of the brain at the tip of the electrode between our algorithm and that done manually by two neurosurgeons, $ICC(3,k)=0.76$, $p<0.001$. When in disagreement, the average distance between regions was 0.82 mm ($\sigma =0.78$), a distance below contact size. Furthermore, it is estimated that electrode contacts electrically sample regions of grey matter within a 3 mm radius. Any discrepancy in identified anatomical regions below this is therefore not clinically significant.

**Table 2:** Validation between manual and automatic segmentation

	Measure	Manual vs automatic	Statistical test	Result
Contact position $N=109/1843$	MAE ($\mu$, $\sigma$, IQR)	M1: 0.38 mm, 0.24, 0.22	Paired *t* test	t (106) = − 1.756, $p=0.82$
		M2: 0.40 mm; 0.22, 0.26	Pearson correlation	$r = 0.454$, $p<0.001$
			Cronbach’s alpha	0.615
	MAE (*x*, *y*, *z* components)	M1: (0.14, 0.15, 0.27) mm		
		M2: (0.17, 0.15, 0.26) mm		
	RMSD	M1: 0.45		
		M2: 0.45		
Bolt angle M1: N=95/224	mean angle difference	M1: 0.59$^\circ$ (1.27)	Paired *t* test	t (94) = − 4.54, $p<0.001$
		M2: 0.22$^\circ$ (1.53)		t (112) = 1.533, $p=0.128$
			Pearson correlation	$r=0.991$, $p<0.001$
				$r=0.985$, $p<0.001$
			Cronbach’s alpha	0.995
				0.992
M2: $N=113/224$	displacement error at first contact due to angle difference ($\mu$, $\sigma$, IQR)	M1: 0.68 mm, 0.81, 0.83	Non-inferiority test	CI = (0.431, 0.926) tolerance = 2.29 mm
		M2: 0.72 mm, 0.84, 0.81		
Regions of anatomy $N=222/222$	region of anatomy at first contact		Intra-class correlation	0.76, $p<0.001$
	distance between regions when in disagreement	0.82 (0.78) mm		

### Bending estimation

In order to study whether Darboux vectors are a representative measure of bending, we look into the relationship (Pearson correlation) between global bending and the following variables: accumulated displacement of contacts ($r=0.532$, $p<0.001$), length of electrode inside the brain tissue ($r=0.373$, $p<0.001$), amount of white matter traversed by the electrode ($r=0.257$, $p<0.001$), and bolt angle ($r=0.189$, $p=0.045$). Of the two surgical approaches, placing a stylet far from TP resulted in larger global bending of electrodes ($\mu =0.49$; $\sigma =0.34$) compared to the bending observed in electrodes that had a stylet placed close to TP ($\mu =0.31$; $\sigma =0.18$), a difference which was statistically significant, $t(222)=5.36$, $p<0.01$.

### Generalisability and robustness

**Fig. 11:**
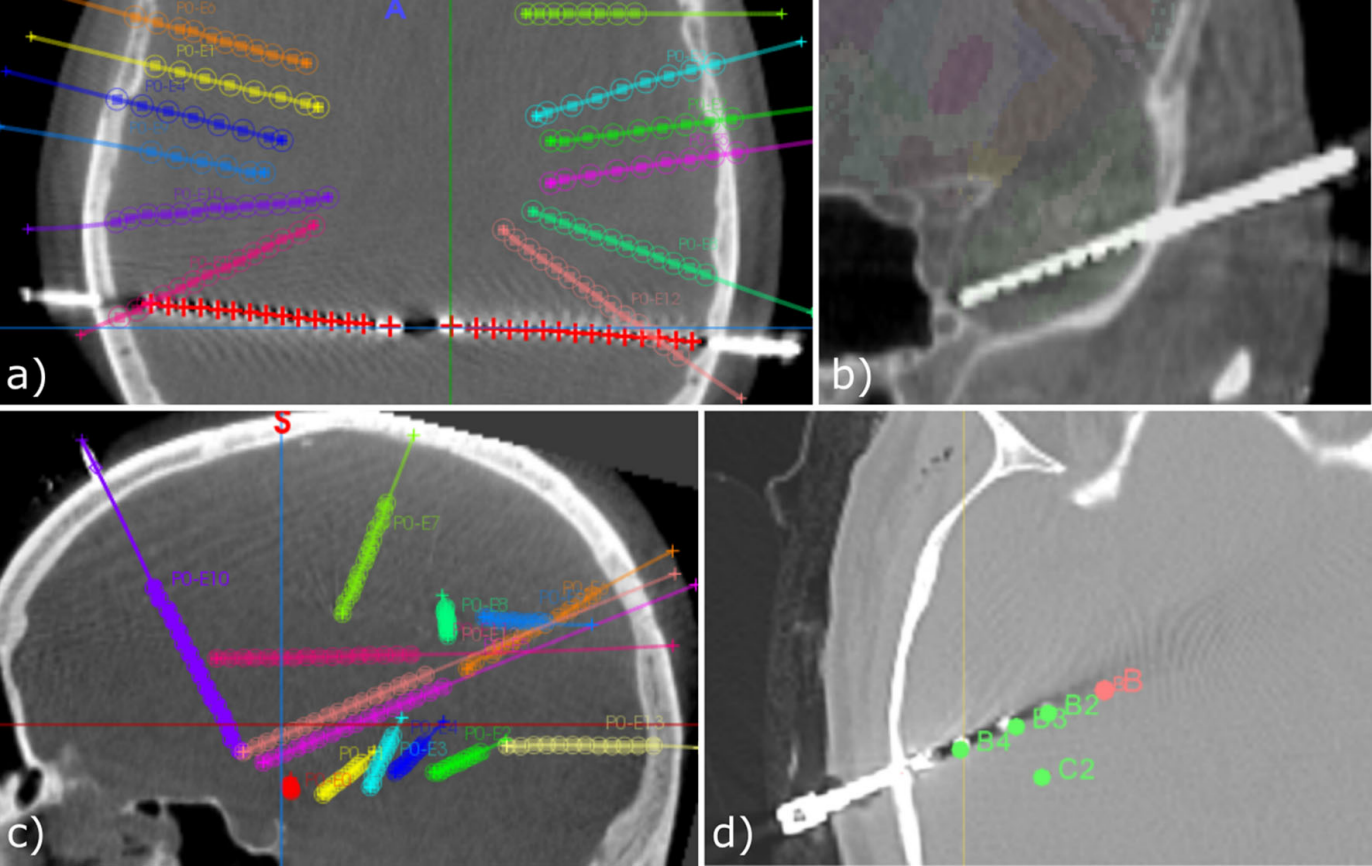
Generalisability and robustness tests. Our proposed algorithm using data from a different centre: **a** with smaller bolt heads, **b** contacts very close to each other, and **c** electrodes inserted deeply (pink electrode with insertion depth of 110 mm); Our data in SEEGA: **d** segmented contact positions (green fiducials) and implantation plan (pink fiducials)

Three SEEG post-resection cases using SEEG DEPTH electrodes (PMT Corp., USA) were obtained from the Vickie and Jack Farber Institute for Neuroscience (Thomas Jefferson University) to assess generalisability and robustness of our algorithm. We observed a reduced average performance (sensitivity = $69.7\%$ and PPV = $82.6\%$) due to the following factors (Fig. 11a–c): (a) smaller bolt heads (our parameter of minimum number of pixels of bolt heads could be adjusted), (b) contacts being very close to each other and merged as single blobs (addressed by adopting optimal oblique resampling used for DBS electrodes [11]), and (c) electrodes inserted deeply (our parameter of maximum electrode length could be adjusted to account for this). Despite this, our algorithm was agnostic of electrode types and implantation planning and was robust in post-resection data sets.

We randomly chose 3 of our data sets to test the method proposed in [2, 16] and implemented in SEEGA (Slicer v4.6.2). We configured electrode types based on electrode specification, used the implantation plan (EP and TP) as fiducials and imported the CT image. We modified SEEGA to use the same threshold that we computed in our algorithm for consistency and because the default threshold computed by SEEGA resulted in segmentations errors. We observed an average sensitivity of $82.9\%$ and PPV of $65.3\%$ ($97.3\%$ and $98.2\%$, respectively, using our algorithm). Whilst we noticed that most of the contact positions were not accurate (Fig. 11d), we only report performance on the number of contacts that were incorrectly or not segmented. Similarly to our approach tested with data from other centres, SEEGA might perform better after fine-tuning parameters. Both algorithms have parameters the user must select to guarantee optimal performance.

## Discussion

### Automatic segmentation

The automatic segmentation of bolts is typically overlooked in the literature and could be used to report accuracy errors caused by differences in angle with respect to planning. We use bold direction to search for contacts with neither prior information of electrode type nor implantation planning. Compared to previous work [2, 14], we use a factor of maximum intensity from CT images rather than a constant. Regions of interests were used to segment position of contacts and bolts based on geometrical properties with their centroids equivalent to the signal peaks found in [11] and more generally in the literature. The choice of intensity threshold and constraints favour few incorrectly segmented contacts (FP) over missing contacts (FN), since these can be easily discarded by surgeons during manual adjustment. We found that FP were located in the inner surface of the skull and were caused by pixel size inaccuracies of CSF regions overlapping with bone structure. The performance of our algorithm is similar to previous approaches although [2] only considers displacements at the tip of the electrode with no details of displacement of other contacts along the electrode and [14] uses a very small sample size assuming rigid electrodes.

Compared to the search strategy by Arnulfo et al. [2], our algorithm uses a higher angle constraint ($30^{\circ }$ rather than $10^{\circ }$) because we use instead the bolt direction to search for contacts rather than a direction from previously segmented contacts. It is also clinically relevant to accurately segment the position of the contacts closer to the skull to ensure grey matter at the cortical entry is adequately sampled, but these might be difficult to segment in the bolt region. Therefore, contacts are predicted in this region after inferring electrode type. Further conditions would need to be included in this step to support more electrodes from different manufacturers. To cope with electrodes crossing, we initially used geometrical features to identify large blobs that relate to more than one contact for splitting. However, the resulting position of contacts was not good enough to make the method fully automatic, so we rely on manual adjustments that can be quickly performed with our interface. Moreover, the reason for contacts not being assigned to an electrode was because of bolt heads or contacts not being segmented and because of incorrectly assigned contacts to bolt heads. However, electrode bending did not influence accuracy of contact assignment as evidenced by our approach performing equivalent between the two surgical approaches (placing a stylet close to and far from TP), where global bending is significantly different.

### Validation

The MAE of the centroids validated from the manual identification of contacts in our study is slightly lower than the localisation error of 0.5 mm reported in [2, 16], although with a greater standard deviation. The RMSD reported in our study of axial and sagittal planes is similar to the RMS reported in [11]. However, we see a higher error in the coronal plane due to greater CT slice thickness ($\mu =0.87$ vs. $\mu =1.14$ mm) and thus a greater RMSD than that reported in their work with deep brain electrodes. We also confirmed the accuracy of the computed contact positions with respect to those from two manual segmentations which varied less that 0.8 mm (CI of $95\%$). We defined equivalence of bolt angles between manual and automatic segmentation as an interval of − 2.29–2.29 mm based on the sample size calculation (14 manual and 14 automatic; power = $90\%$, $p<0.05$, $\sigma =1$ mm) for the angle error displacement. The sample size has also been increased to account for the possible effect of clustering of electrodes within patients, with an assumed $ICC=0.25$ and average number of 8 electrodes per patient. The mean angle difference observed (paired *t* test) is small and has a strong and positive correlation and good reliability. Related to the non-inferiority test of the displacement error caused by this angle difference, there is no suggestion that, at the tolerance level of contact length, either method is worse that the other. We were also able to confirm that the anatomy regions at the tip of the electrodes are concordant with those manually identified by two neurosurgeons. Our sample size is above the sample size computed (159) that is sufficient for a 95% confidence interval with width $\pm \,0.1$ assuming an estimate of 0.6 ICC. This is important for post-surgical analysis of SEEG electrodes as knowing the anatomical region each contact is located in can aid in identifying the seizure onset zone.

### Bending

Compared to previous approaches for DBS that fit trajectories along electrodes using polynomials [10, 11], we quantify the amount of bending as well as the displacement at contact positions, permitting to study the reasons of bending within the anatomy. The parcellation is used to accurately report the anatomical regions the electrodes have traversed. We were able to estimate local bending by modelling electrodes as elastic rods and using Darboux vectors to quantify the 3-degrees-of-freedom rate of change of the material frames orthogonally aligned to the electrode. The large and positive correlation observed between global bending and the accumulated displacement of contacts in addition to the medium positive correlation with length of electrode indicate that Darboux vectors are a representative measure of bending. The projection of a rigid rod is based on the bolt direction rather than on planned trajectories as in previous studies. This facilitates evaluating the displacement of each contact, rather than a displacement due to EP location errors and angle of drilling. We confirmed the displacement at the tip of the electrode due to angle difference between bolts automatically and manually segmented is below a tolerance displacement error, and therefore, we were able to report contact displacement due to bending with respect to a rigid electrode.

## Conclusions and future work

We present a method for automatic segmentation of electrodes, including their contacts and bolts, that takes bending into account by quantitatively estimating local and global bending. We show the importance of accurately detecting the angle of the bolt, since it is one of the main reasons for TP errors, as well as the importance of accurately and automatically reporting the region of anatomy the contacts are located in, since it aids identifying the seizure onset zone. Our approach was validated in 23 data sets comprising two surgical techniques and demonstrated in these cases our method is robust to bending along the electrode.

Future work is required to guarantee generalisability of automatic segmentation of SEEG electrodes by enabling automatic parameter selection to support data from multiple centres. We hypothesise that white matter tracks may be one of the factors of electrodes bending, and therefore, we envisage using diffusion MRI tractography in combination with our proposed methods in future studies to understand the reasons to bending. Understanding the mechanical properties of electrodes along with the biomechanical properties of the brain tissue as well as simulating instrument–tissue interaction will permit greater fidelity to the implantation plan resulting in more accurately targeting specific regions and potentially improve clinical outputs including the ability to reduce the number of implanted electrodes and targeting riskier areas. We envisage to incorporate our work to an EEG analysis pipeline and validate the activity read from SEEG contacts with their anatomical location. Parallel clinical work will look into different types of techniques and their effect on electrodes bending, i.e. understanding the reasons why pushing a stylet closer to the target point result in lower bending of electrodes.

## Electronic supplementary material

Below is the link to the electronic supplementary material.

Supplementary material 1 (wmv 8382 KB)

## Footnotes

1 https://github.com/InteractiveComputerGraphics/PositionBasedDynamics.

2 http://mitk.org.

3 http://itk.org/.
